# Occupational Therapists, Physiotherapists and Orthopaedic Surgeons Agree on the Decision for Carpal Tunnel Surgery

**DOI:** 10.34172/ijhpm.2020.227

**Published:** 2020-12-16

**Authors:** Karina J. Lewis, Michel W. Coppieters, Bill Vicenzino, Ian Hughes, Leo Ross, Annina B. Schmid

**Affiliations:** ^1^Occupational Therapy Department, Gold Coast University Hospital, Southport, QLD, Australia.; ^2^Menzies Health Institute Queensland, Griffith University, Brisbane and Gold Coast, QLD, Australia.; ^3^Amsterdam Movement Sciences, Faculty of Behavioural and Movement Sciences, Vrije Universiteit Amsterdam, Amsterdam, The Netherlands.; ^4^School of Health and Rehabilitation Sciences: Physiotherapy, The University of Queensland, Brisbane, QLD, Australia.; ^5^Office of Research Governance and Development, Gold Coast University Hospital, Southport, QLD, Australia.; ^6^Division of Allied Health, Queen Elizabeth II Jubilee Hospital, Brisbane, QLD, Australia.; ^7^Nuffield Department of Clinical Neurosciences, University of Oxford, Oxford, UK.

**Keywords:** Advanced Scope Practice, Occupational Therapy, Physiotherapy, Hand Therapy, Carpal Tunnel Syndrome

## Abstract

**Background:** Therapist-led pathways have been proposed as waitlist management strategies prior to surgery for conditions such as carpal tunnel syndrome (CTS) in public hospitals. These models of care typically shift the initial care of patients and decision-making from surgeons to therapists and, have been shown to reduce the number of patients requiring surgery and improve wait-times. This occurs despite limited evidence of surgeon-therapist agreement on key decisions, such as the need for surgery. The purpose of this was study was to assess the agreement between therapists and orthopaedic surgeons regarding the need for surgery for patients who have CTS.

**Methods:** This blinded inter-rated agreement study was embedded in a multicentre randomised parallel groups trial of 105 patients with CTS referred to four orthopaedic departments and waitlisted for an appointment. The trial evaluated the effect of a therapist-led care pathway on the need for surgery and outcomes related to symptoms and function. Patients were randomised to either remain on the orthopaedic waitlist or receive group education, a splint and home exercises. The decision on the need for surgery at 6 months was made by a member of the orthopaedic consultant team or by one of the 14 participating therapists. The therapists and surgeons were blinded to each other’s decision. Agreement was determined using percentage agreement, kappa coefficients (k), prevalence-adjusted and bias-adjusted kappa (PABAK), and Gwet’s first-order agreement coefficient (AC1).

**Results:** Substantial agreement was seen between therapists and surgeons regarding the need for surgery (PABAK=0.74 (0.60-0.88)). Agreement was significantly associated with experience (*P*=.02). Therapists with advanced experience and scope of practice demonstrated perfect agreement with surgeons (PABAK=1.00 (95% CI: 1.00-1.00)). Mid-career therapists demonstrated substantial agreement (PABAK=0.67 (95% CI: 0.42-0.91)) and early-career therapists demonstrated fair agreement (PABAK=0.43 (95% CI: -0.04-0.90)).

**Conclusion:** Therapists with advanced scope of practice make decisions that are consistent with orthopaedic surgeons.

## Introduction

**Key Messages**

** Implications for policy makers**

- Therapists and surgeons make comparable decisions on the need for carpal tunnel surgery.
- Therapists with advanced experience and training agree more closely to that of surgeons on the need for carpal tunnel surgery.
- This study supports the use of therapist-led, advanced practice models of care for the management of carpal tunnel syndrome (CTS) in the public hospital system.

** Implications for the public**

To manage growing patient numbers and waitlists, health services are employing therapists in an advanced scope of practice to manage musculoskeletal conditions. Our research suggests that therapists make similar decisions to that of surgeons regarding the need for surgery for carpal tunnel syndrome (CTS). CTS is one of the most common conditions on orthopaedic waitlists. The substantial agreement on the need for surgery between therapists and surgeons together with the previously reported ability to reduce the need for surgery^1^ provides support for therapist-led models of care implemented prior to surgery for patients with CTS in public health settings. The data can be used when implementing therapist-led models of care of patients who have CTS.

Carpal tunnel syndrome (CTS) is a common upper limb neuropathy^2^ caused by compression of the median nerve at the wrist. CTS accounts for at least 10% of patients referred to public orthopaedic departments and the surgical management of CTS is one of the most commonly performed elective surgical procedures in public hospitals.^3^ Despite evidence of significant socioeconomic burden,^4-6^ CTS is typically considered a low priority for access to specialist outpatient appointments and surgery in public hospitals.^7^ As such, wait-times for outpatient consultation and surgery for CTS are often long, presenting a significant barrier for access to care within public health systems.^3^ This is of significance as longer wait times are associated with poorer clinical outcomes and patient satisfaction post-operatively.^8,9^ With the number of CTS surgeries predicted to double over the coming decade,^10^ planning and development of adequate workforce and service delivery models to manage wait times are of importance.

 To manage growing patient numbers, health services are increasingly adopting novel models of care as a strategy to improve treatment access.^11-14^ For instance, triage clinics and therapist-led models of care, where therapists independently make treatment decisions within an agreed clinical decision-making framework, have been implemented for the management of musculoskeletal conditions.^11,14-19^ Results of retrospective and prospective studies suggest that these models of care have a positive impact on accelerating access to care, reducing the rate of surgery (if implemented prior to surgery) and improving patient outcomes and satisfaction.^1,15,16,20^

 In these models of care, clinicians are required to independently assess, provide patient education and therapeutic interventions as well as make treatment decisions that are considered within, but more advanced than their typical scope of practice (for example, assessing the need for surgical intervention). Assurance of the safety and quality of practice regarding therapist-led models of care has been identified as a concern by key stakeholders.^21^ Additionally, recommendations have been made for further assessment of both the clinical decision-making process and governance frameworks supporting therapist-led models of care.^22,23^ Previous studies have demonstrated high agreement between physiotherapists working within a therapist-led model of care and orthopaedic surgeons regarding the need for surgical management of conditions of the shoulder,^24,25^ hip and knee.^18,19^ There are no published data relating to the agreement between therapists and surgeons for the need for surgery for common hand conditions, such as CTS. Given therapist-led models of care are being proposed as a waitlist management strategy for hand conditions,^1,14,16^ it is important to determine if therapists make decisions regarding a patient’s need for surgery that are consistent with surgeons to support these service developments.

## Purpose of the Study

 The purpose of this study was to assess the level of agreement between therapists and orthopaedic surgeons regarding the need for surgery for patients with CTS.

## Methods

 This study was embedded within a larger, multicentre randomised clinical trial that aimed to determine the efficacy of a therapist-led model of care for patients with CTS.^1,26^

 The study was undertaken within the outpatient setting of 4 Queensland Health public hospitals in Australia (Queen Elizabeth II Jubilee Hospital [Brisbane], Logan Hospital [Logan]; Rockhampton Hospital [Rockhampton]and Gold Coast University Hospital [Southport]). All patients provided written informed consent before participating in the trial. Reporting of this agreement study has been based on recommendations by Kottner et al.^27^

###  Patient Participants

 Patients aged between 18 and 75 years with a clinical diagnosis of CTS confirmed by nerve conduction studies (NCS) were recruited from waitlists for orthopaedic consultation. NCS were completed prior to referral to the participating hospitals as per standard referral processes. Inclusion criteria comprised the presence of CTS symptoms for longer than 2 months, the ability to comprehend the requirements of the study and provide consent. Exclusion criteria included CTS related to recent trauma or pregnancy, osteoarthritis of the wrist or hand, other musculoskeletal or neurological conditions affecting the upper limb, and systemic conditions (other than diabetes). Patients with pending litigation or insurance claims, those who received a steroid injection within the previous 6 months or hand therapy interventions within the previous 3 months (orthosis or exercises) were also excluded. These exclusion/inclusion criteria were defined for the original randomised clinical trial^1,26^ and were designed to include patients with idiopathic CTS while minimising confounding factors that may influence hand symptoms and function. Recruitment for the agreement study commenced in April 2014 and ceased in April 2017.

###  Therapist-Led Model of Care

 Eligible patients were randomly allocated to the experimental group (education, splinting (orthosis) and a home exercise program via a therapist-led care model), or control group (remaining on the waitlist without additional care). The trial protocol^26^ and findings^1^ have been published elsewhere. For this agreement study, all patients were independently seen by an orthopaedic surgeon and a therapist 6 months after randomisation to determine the need for surgery.

###  Clinician Assessors

 Patients were seen by a member of the orthopaedic surgery team, which consisted of interns, residents, orthopaedic registrars, and consultant orthopaedic surgeons. For those seen by an intern or resident, the patient’s presentation was either discussed with, and/or the patient was also reviewed by a registrar or consultant to determine the need for surgery as per standard hospital processes. For the purpose of this study, the medical doctor/s involved in the decision regarding the need for surgery will be referred to as the orthopaedic team.

 Therapists involved in the study consisted of 12 occupational therapists and 2 physiotherapists (Table 1). To assist with consistency of practice, all participating therapists worked within a therapist-led model of care framework. In addition to the trial protocol, therapists were required to read textbook chapters relating to the management of CTS.^28-30^ Therapists were required to observe a member of the research team complete the required clinical examinations, interventions and outcome measures. Senior therapists and/or the site coordinator were available to provide guidance relating to patient care or research protocol if required.

**Table 1:** Clinician Assessors Experience by Profession

	**Occupational Therapist**	**Physiotherapist**	**Total**
	**No. (%)**	**No. (%)**	**No. (%)**
Early-career	3 (21)	0 (0)	3 (21)
Mid-career	4 (29)	1 (7)	5 (36)
Advanced	5 (36)	1 (7)	6 (43)

###  Outcome Measures

 Patient characteristics such as demographics, symptom duration and nerve conduction study results were collected. Patient-reported symptom severity, satisfaction and perceived changes in symptoms were also collected as part of the clinical trial and are detailed in full elsewhere.^1^

###  Primary Outcome Measure

 The primary outcome was the decision about the need for surgery made by therapists (within the therapist-led model of care framework) and the orthopaedic team. These decisions were made independently at 2 different appointments between the patient and the therapist, and the patient and the orthopaedic team. These appointments occurred at the end of the follow-up period (ie, 6 months following randomisation) for patients from both the control and experimental groups.

 In order to make an independent clinical recommendation regarding the need for surgery, the therapists as well as the orthopaedic team had access to the patient’s medical record and NCS results. Patient history was taken, and clinical assessments were completed by both the orthopaedic team and therapist. Both assessors completed a paper assessment form with the question *‘If CTS symptoms and the ability for this patient to use their hands stay as they are now, are you likely to recommend surgery?’* The paper form included a multiple-choice answer box with answer choices of ‘yes,’ ‘no,’ and ‘not applicable’ for both left and right hands.’ A box for ’not applicable’ was included to accommodate patients who had only ever experienced unilateral symptoms and NCS was negative for CTS in the same hand (and therefore surgery had never been a consideration). In the case of bilateral symptoms, only one hand, which was pre-determined by flip of a coin prior to randomisation, was included in the analysis.

###  Secondary Outcome Measure

 We evaluated the experience of therapists according to years of postgraduate practice and advanced hand therapy qualifications. For the purpose of this study, we categorised the therapists’ experience into advanced (10 or more years), mid-career (2-9 years) and early-career (less than 2 years). As the study ran over 3 and a half years, therapists’ full-time equivalent years of experience was expressed as an average based on when the first and last decision was made by an individual therapist. In addition, we also recorded the completion of advanced postgraduate qualifications (eg, Accredited Hand Therapists as awarded by the Australian Hand Therapy Association or Certified Hand Therapists as awarded by the Hand Therapy Certification Commission, USA).

 Similar data regarding the number of orthopaedic doctors and level of experience were not obtained as it is routine practice for junior doctors to discuss patients with more senior members of the orthopaedic team. Additionally, the orthopaedic teams’ opinion on whether surgery was or was not required was chosen as the ‘reference standard’ for this analysis given doctors are typically the primary decision-makers in determining the need for surgery within the healthcare team.

###  Order of Therapist and Surgeon Examinations and Blinding

 Due to therapist and orthopaedic team schedules and clinic times, patients were routinely booked to see the therapist first. On the same day, following the therapy appointment, patients attended an appointment with the orthopaedic team. If the patient was unable to wait or if other scheduling conflicts existed, the patient was booked for their orthopaedic appointment within 2 weeks of their therapist appointment. This was deemed an acceptable timeframe given that CTS is a chronic condition and symptoms are unlikely to change significantly over a 2-week period.^31^ Both therapists and orthopaedic team were blind to each other’s assessment and decision regarding the need for surgery.

 Two therapists were involved with each patient, one being responsible for completion of the outcome measures associated with the concurrent clinical trial, who was blinded to group allocation. The other was responsible for providing the treatment based on group allocation and therefore was unblinded. Either therapist could make the decision regarding the need for surgery based on convenience. The decision was not discussed with other team members.

###  Data Analysis

####  Analysis and Sample Size

 Data were analysed using Stata 14.2 (College Station, TX, USA) and Microsoft Excel (Microsoft Corp. 2018). The study was powered to detect group differences in the original clinical trial. Eighty-five patients had recorded recommendations regarding surgery from both a therapist and an orthopaedic team. This sample size was deemed sufficient to detect a kappa of 0.61 with a 95% confidence interval of 0.18 based on an estimated 66% being recommended for surgery.^26^ This kappa value reflects substantial agreement according to Landis and Koch.^32^ This precision was estimated using the ‘sskdlg’ package of Stata and based on the procedure of Cantor.^33^ Our sample size is similar to other agreement studies.^19,25,34^

 Descriptive statistics were used for baseline characteristics. Agreement measures used were proportion agreement, Cohen’s kappa coefficient (k),^35^ prevalence-adjusted and bias-adjusted Kappa (PABAK)^36^ and Gwet’s first-order agreement coefficient (AC1).^37^ Cohen’s kappa coefficient is known to be adversely affected by prevalence and bias which was present in the analysis of early-career therapist decisions in this paper. Gwet’s AC1 and PABAK were therefore also used in the analysis of this study. In addition, we report Cohen’s kappa coefficients to allow comparison with previous musculoskeletal agreement studies.^24,25^ A Kappa/PABAK/Gwet’s AC1 value of 0 indicates the observed agreement is by chance only. Positive values suggest greater than chance agreement. Strength of agreement was assessed for each measure as per the scale suggested by Landis and Koch^38^: 0.2 or less = poor agreement, 0.21-0.4 = fair agreement, 0.41-0.6 = moderate agreement, 0.61-0.8 = substantial agreement, 0.81-1 = near perfect agreement.

 Logistic regression was used to test the effects of experience of the therapists and group allocation (treatment vs control group) of the patients on agreement (both recommending surgery or non-surgery).

## Results

 In the clinical trial, 105 patients with CTS were randomised. A decision for surgery by both the surgeon and therapist was available for 85 patients (Figure). Patient characteristics are described in Table 2. Data regarding the therapist’s recommendation for surgery was not recorded for ten patients despite them attending their appointment with the therapist. Demographic and clinical summary data is provided for these individuals alongside similar data for those participants who were included in the agreement analyses (Table 2). Five patients failed to attend their therapy appointment. Three patients failed to attend both therapist and orthopaedic appointments. One patient failed to attend their appointment with the orthopaedic team and one patient underwent surgery at another hospital prior to their 6-month review.

**Figure:**
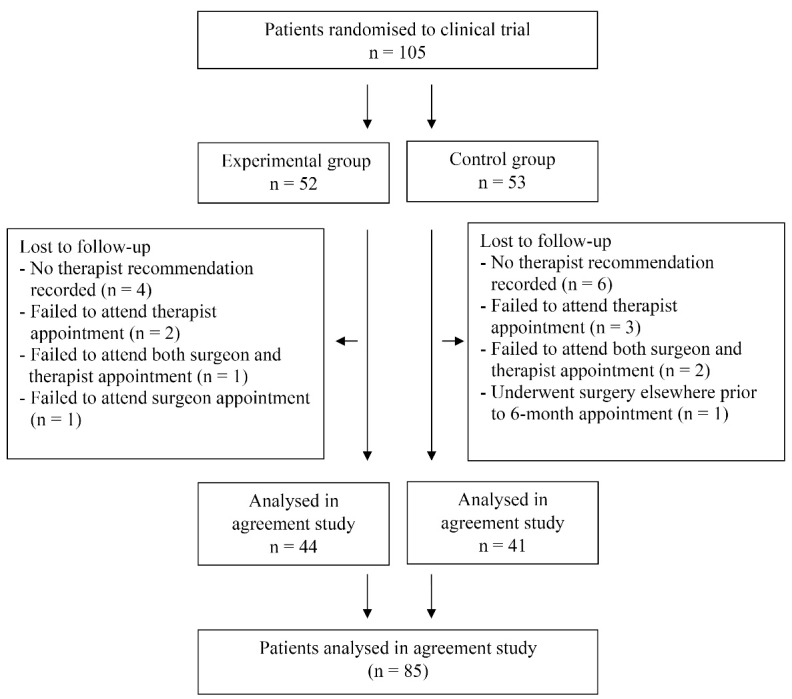
Study Diagram.

**Table 2:** Patient Characteristics

**Characteristics**	**Total (n = 85)**	**Missing Therapist Decision Data (n = 10)**
Mean (SD) age in years	51.0 (12.3)	46.1 (13.7)
Mean (SD) duration of symptoms in years	4.0 (4.5)	4.1 (3.1)
Number (%) female	55 (64.7)	9 (90.0)
Undertaking paid employment, No. (%)	57 (67.1)	7 (70.0)
Presence of any medical co-morbidity, No. (%)	70 (82.6)	9 (90.0)
Presence of thenar wasting, No. (%)	13 (15.3)	3 (30.0)
Mean (SD) Boston Symptom Severity Questionnaire	2.7 (0.8)	3.1 (0.5)
Mean (SD) Boston Function Severity Questionnaire	2.3 (0.9)	2.2 (0.6)
Nerve conduction study (0-6)		
0: Normal, No. (%)	0 (0.0)	0 (0.0)
1: Very mild, No. (%)	2 (2.4)	0 (0.0)
2: Mild, No. (%)	28 (32.9)	2 (20.0)
3: Moderate, No. (%)	35 (41.2)	5 (50.0)
4: Severe, No. (%)	8 (9.4)	2 (20.0)
5: Very severe, No. (%)	11 (12.9)	1 (10.0)
6: Extremely severe, No. (%)	1 (1.2)	0 (0.0)

Abbreviation: SD, standard deviation.

 There was substantial agreement (PABAK = 0.74, CI: 0.60-0.88) regarding the need for surgery between therapists and orthopaedic team (74/85 patients; agreement for surgery: 51 patients; agreement for no surgery: 23 patients). In 5 patients, the orthopaedic team recommended surgery and the therapist did not, in 6 patients the orthopaedic team did not recommend surgery whereas the therapist did recommend surgery (Table 3).

**Table 3:** Agreement Regarding the Need for Surgery in All therapists, Early-Career Therapists, Mid-Career therapists and Advanced Therapists

		**Surgeons**		
		**Surgery**	**No Surgery**	**Total**
All therapists	Surgery	51	6	57
	No surgery	5	23	28
	Total	56	29	85
Early-career therapists	Surgery	10	2	12
	No surgery	2	0	2
	Total	12	2	14
Mid-career therapists	Surgery	17	3	21
	No surgery	2	13	15
	Total	19	17	36
Advanced therapists	Surgery	16	0	16
	No surgery	0	7	7
	Total	16	7	23

 According to years of practice, 6 therapists were at an advanced stage of their career, 5 mid-career and 3 early-career. Five therapists in the advanced group and one in the mid-career group were Accredited Hand Therapists (as awarded by the Australian Hand Therapy Association) or Certified Hand Therapists (as awarded by the Hand Therapy Certification Commission, USA). Therapist experience (by years) had a significant impact on agreement (odds ratio = 3.8, *P* = .02) (Table 3). Decisions made by advanced therapists demonstrated perfect agreement with surgeons (k = 1.00). Decisions made by mid-career practitioners demonstrated substantial agreement (k = 0.66) and decisions made by early-career practitioners demonstrated poor agreement when considering the kappa score alone (k = -0.17, CI: -0.35-0.02). However, the latter kappa value should be viewed with caution as it is unduly affected by the low prevalence of non-surgery recommendations for this sub-group (all but 2 patients were recommended for surgery by the orthopaedic surgeon). To allow comparison between early-career, mid-career and advanced therapists, PABAK and Gwet’s analysis was completed. When adjusted for this low prevalence, the resulting PABAK score for the early-career therapists was 0.43 (fair agreement) and Gwet’s AC1, which is refractory to low prevalence effects,^39^ was 0.62 (substantial agreement, Table 4). Analyses categorising therapists according to advanced qualifications revealed comparable results (Supplementary file 1, Tables S1 and S2).

**Table 4:** Agreement Coefficients Between Orthopaedic Team and Therapists Regarding the Need for Surgery

**Recommendation for Surgery**	**Kappa**	**PABAK**	**Gwet’s AC1**	**% Agreement**
	**(95% CI)**	**(95% CI)**	**(95% CI)**	**(95% CI)**
All therapists	0.71 (0.55-0.87)	0.74 (0.60-0.88)	0.77 (0.63-0.90)	0.87 (0.80-0.94)
Early-career	-0.17 (-0.35-0.02)	0.43 (-0.06-0.92)	0.62 (0.16-1.00)	0.71 (0.44-0.99)
Mid-career	0.66 (0.41-0.92)	0.67 (0.42-0.91)	0.67 (0.41-0.92)	0.83 (0.71-0.96)
Advanced	1.00 (1.00-1.00)	1.00 (1.00-1.00)	1.00 (1.00-1.00)	1.00 (1.00-1.00)

 Abbreviations: PABAK, prevalence-adjusted and bias-adjusted kappa; AC1, first-order agreement coefficient.

 The patients’ allocated treatment group within the overarching clinical trial did not influence proportion agreement (odds ratio = 1.34 CI (0.37-4.77), *P* = 0.65) and agreement coefficients were similar between control and experimental groups (kappa: 0.65 versus 0.76, *P* = 0.51; PABAK: 0.71 versus 0.77, *P* = 0.66; Gwet’s AC1: 0.75 versus 0.79, *P* = 0.80).

 No serious or unexpected adverse events were reported in the study. Nine participants in the experimental group reported mild and transient symptoms that are detailed elsewhere.^1^ No safety issues related to the therapist-led care pathway were identified by departmental leads (allied health and orthopaedics) or reported by the clinical assessors. No patient complaints were received.

## Discussion

 Our results demonstrate substantial agreement between therapists and orthopaedic surgeons regarding the need for surgery for patients with CTS. Therapists’ experience was positively associated with proportion agreement, indicating that advanced therapists align better with the orthopaedic team’s decision-making. It is worth noting that more junior therapists still made decisions with a reasonable level of agreement, even without the clinical input of a more senior therapist as was the case for this study protocol. In everyday practice at sites where these models of care are in use, it is usual for more junior therapists to seek advice and input on decision-making from more advanced therapists, which could further improve the reliability of their decision-making. This is consistent with how usual care in the medical model operates around the supervision of junior doctor practice and decision-making.

 Therapist-led models of care including non-surgical management such as education, splinting and exercises delivered prior to surgery have shown to improve access to care and reduce the need for surgery.^1,14,16^ These models are not only likely to reduce costs and waiting time, but may also have added benefit for patients such as a reduction of multiple contact points and duplication in health services (eg, through concurrent provision of rehabilitation advice and surgical recommendation).^40^ The lack of data regarding the clinical decision-making processes of therapists working within therapist-led models of care have been a limiting factor for their more widespread implementation.^23^ Our findings of comparable agreement between experienced therapists and the orthopaedic team regarding the need for CTS surgery contributes to the evidence supporting therapist-led models of care.

 Clinical guidelines for CTS endorsed by professional surgical associations recommend surgery for patients where non-surgical options have failed, where symptoms are severe or prolonged, or if the patient wishes to pursue surgery based on the symptoms they are experiencing.^4^ As in previous studies examining the usefulness of therapist-led clinics,^24,25^ we used the orthopaedic team’s opinion regarding need for surgery as a ‘reference standard’ to compare therapists decisions. Although it remains unclear which decision was in fact the best decision for individual patients, this practice reflects traditional care models. Additionally, patient preference would usually be a significant factor influencing the decision regarding the need for surgery. To minimise this influence (given it was not within the therapist scope of practice to book and consent for surgery) and in absence of a standardised outcome measure, we asked the clinical assessors (therapists and surgeons) for their recommendation regarding the need for surgery, not necessarily the final outcome of the appointment.

 Our results of perfect agreement regarding the need for surgery between advanced therapists and the orthopaedic team are in line with previous publications assessing agreement between advanced practice physiotherapists and orthopaedic surgeons for other musculoskeletal conditions of the hip, knee and shoulder.^18,19,24,25^ As these studies only involved one advanced physiotherapist, no inferences about the effect of experience on these decisions could be made. One study did show substantial agreement regarding the need for knee surgery between one physiotherapist with one year clinical experience and physicians,^41^ which revealed a higher agreement than our study for the equivalent experience. No studies were found assessing the agreement between occupational therapists and orthopaedic surgeons.

 The finding of higher agreement between surgeons and advanced rather than early or mid-career therapists is an important consideration in the future development of therapist-led models of cares. We would like to point out though that the relatively large confidence intervals of the early-career group (by years of experience) suggest that this estimate is imprecise and is likely a function of the low numbers in this group. Nevertheless, defining the therapist experience according to advanced qualifications confirmed that experienced therapists had higher agreement with the orthopaedic team. Workforce sustainability relating to the availability of advanced staff (particularly in rural and remote areas), budget requirements, and the time and resources required to develop advanced skills, has been identified as a potential barrier to the long-term success of therapist-led models of care.^42^ Provision and support of therapist training pathways and the implementation of keystakeholder-endorsed practice frameworks which support safe decision-making by less experienced staff, while increasing their capability could assist with addressing these challenges in workforce sustainability. In locations where an advanced therapist may be unavailable, such practice frameworks could benefit from remote mentorship to supervise junior therapists. This concept of peer supervision is not dissimilar to the current practice of junior doctors being supervised by registrars and consultants working together in a team. This also supports the desire to develop a more holistic and sustainable workforce model for implementing and managing these models of care into the future.

 This study has certain limitations. Firstly, patients were required to have undergone NCS prior to referral to the participating hospitals. The necessity of NCS to diagnose CTS is heavily debated^43,44^ particularly in relation to their limited sensitivity and specificity^45^ and poor correlation with clinical symptoms^46^ In our research, the NCS helped to exclude conditions that present with similar symptoms (such as cervical radiculopathy and systemic peripheral neuropathy).^47^ In addition, the performance of NCS was standard practice at participating hospitals and the benefit of a confirmed diagnosis was therefore afforded to both therapists and the orthopaedic team. The use of NCS in the diagnosis of CTS in our study setting may have reduced the complexity of patient management and may therefore limit the generalisability of results to other settings where NCS is not performed. Secondly, in 10 cases, there were omissions on the part of the therapist clinical assessor in the completion of the outcome measure. Even though the patients with missing data appeared similar to included patients, it remains possible that missingness was not random. We do not know the reason for these omissions. For example, whether the omission was due to an administrative oversight by the responsible therapist or missed due to unclear decision-making processes regarding the need for surgery by the therapist. Thirdly, we found that experience contributed to higher agreement, which suggests that therapists with advanced experience are best placed to lead these care models. A caveat in inferring more broadly from these findings is the way advanced experience is categorised. Whereas we used years of practice, an alternative way to categorise experience could be the completion of additional relevant qualifications. Of note, these 2 ways of classification were closely aligned in our cohort. Five of the 6 advanced therapists with ten or more years’ experience were also either Accredited Hand Therapists (Australian Hand Therapy Association) or Certified Hand Therapists (USA) whereas only one in the mid-career group had this qualification. Our supplementary analyses categorising therapist experience according to additional qualifications thus corroborated our finding that more experienced therapists showed better agreement than less experienced therapists regarding the need for CTS surgery (Tables S1 and S2). It remains to be shown whether it is the years of experience or additional qualifications that improve agreement. Finally, apart from years of experience for therapists, we did not collect descriptive data on either the therapist or orthopaedic clinical assessors who made the decision on surgery. This might be a limitation in making inferences to the implementation or application of our findings.

## Conclusion

 Our findings show substantial agreement regarding the recommendation of CTS surgery between therapists and orthopaedic surgeons, with the more experienced therapists showing better agreement. These findings provide important information on guiding the implementation of therapist-led models of care as a management strategy for people with CTS in public health settings.

## Acknowledgements

 The authors would like to thank all participants as well as clinicians involved in this trial.

## Ethical issues

 Approval was gained from relevant ethics review boards prior to commencement (HREC/13/QPAH/434).

## Competing interests

 Authors declare that they have no competing interests.

## Authors’ contributions

 ABS, LR, MWC, and KJL conceptualised and designed the study. KJL, LR, MWC, and ABS acquired funding. The study was coordinated by KJL. Data were analysed by KJL and IH, and interpreted by KJL, IH, ABS, BV, and LR. KJL, ABS, IH, BV, LR, MWC wrote the manuscript and KJL prepared figures and tables. All authors provided intellectual input in the various manuscript drafts and approved the final version.

## Disclaimer

 The views expressed are those of the authors and not necessarily those of the NHS, the NIHR or the Department of Health. The funders of the study had no role in study design, data collection, data analysis, data interpretation, writing of the report or decision to publish.

## Funding

 The study was supported by a Health Practitioner Stimulus Grant from Queensland Health and a Small Project Investment Grant of the Private Practice Trust Fund (PPTF) of Gold Coast Hospital and Health Service. ABS is supported by the National Institute for Health Research (NIHR) Biomedical Research Centre (BRC) Oxford.

## Supplementary files

Supplementary file 1 contains Tables S1-S2.
