# Supplementary material for: Occupational Therapists, Physiotherapists and Orthopaedic Surgeons Agree on the Decision for Carpal Tunnel Surgery
Source: Int J Health Policy Manag. 2020 Dec 16;11(7):1001–8. doi: 10.34172/ijhpm.2020.227 (PMC9808184; doi:10.34172/ijhpm.2020.227)
Supplement: Supplementary file 1 — contains Tables S1-S2. [file ijhpm-11-1001-s001.pdf]

Supplementary file 1

Table S1. Agreement regarding the need for surgery based on hand therapy specialisation qualification A: No hand therapy qualification; B: Hand therapy qualification (Accredited Hand Therapist (AHTA, Australia)/Certified Hand Therapist (CHT, USA))

**A**		Surgeon		
		Surgery	No surgery	Total
No advanced hand therapy qualification	Surgery	34	6	40
	No surgery	4	14	18
	Total	38	20	58

**B**		Surgeon		
		Surgery	No surgery	Total
Advanced hand therapy qualification (AHT/CHT)	Surgery	9	0	9
	No surgery	0	6	6
	Total	9	6	15

Table S2. Agreement coefficients for the level of agreement between surgeons and therapists with and without specialist hand therapy qualifications (Accredited Hand Therapist (AHTA, Australia)/Certified Hand Therapist (CHT, USA)) regarding the need for surgery.

Recommendation for surgery	Kappa (95% CI)	PABAK (95% CI)	Gwet's AC1 (95% CI)	Proportion Agreement (95% CI)
All therapists	0.71 (0.55-0.87)	0.74 (0.60-0.88)	0.77 (0.63-0.90)	0.87 (0.80-0.94)
No AHT/CHT	0.61 (0.55-0.82)	0.65(0.46-0.85)	0.69 (0.50-0.88)	0.83 (0.73-0.93)
AHT/CHT	1.00 (0.52-1.00)	1.00 (1.00-1.00)	1.00 (1.00-1.00)	1.00 (1.00-1.00)
